# Diastereoselective and enantioselective reduction of tetralin-1,4-dione

**DOI:** 10.3762/bjoc.4.37

**Published:** 2008-10-22

**Authors:** E Peter Kündig, Alvaro Enriquez-Garcia

**Affiliations:** 1 Department of Organic Chemistry, University of Geneva, 1211 Geneva 4, Switzerland

**Keywords:** asymmetric, catalysis, ketone, reduction, tautomer

## Abstract

**Background:**

The chemistry of tetralin-1,4-dione, the stable tautomer of 1,4-dihydroxynaphthalene, has not been explored previously. It is readily accessible and offers interesting opportunities for synthesis.

**Results:**

The title reactions were explored. L-Selectride reduced the diketone to give preferentially the *cis*-diol (d.r. 84 : 16). Red-Al gave preferentially the *trans*-diol (d.r. 13 : 87). NaBH_4_, LiAlH_4_, and BH_3_ gave lower diastereoselectivities (yields: 76–98%). Fractional crystallization allowed isolation of the *cis*-diol and the *trans*-diol (55% and 66% yield, respectively). Borane was used to cleanly give the mono-reduction product. Highly enantioselective CBS reductions afforded the *trans*-diol (72% yield, 99% ee) and the mono-reduction product (81%, 95% ee).

**Conclusion:**

Diastereoselective and enantioselective reductions of the unexplored tetralin-1,4-dione provides a very convenient entry into a number of synthetically highly attractive 1,4-tetralindiols and 4-hydroxy-1-tetralone.

## Introduction

In this article, we briefly review synthetic approaches to 2,3-dihydro-1,4-naphthoquinone, more simply named tetralin-1,4-dione (**2**). This symmetric diketone is the stable tautomer of 1,4-dihydroxynaphthalene (**1**). Although known for many years, it has never been used in synthesis. The reactions of **2** that are reported in this article are those given in the title.

**Scheme 1:**
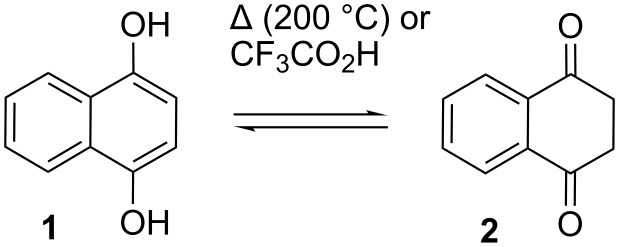
Tautomerization of 1,4-dihydroxynaphthalene.

**Scheme 2:**
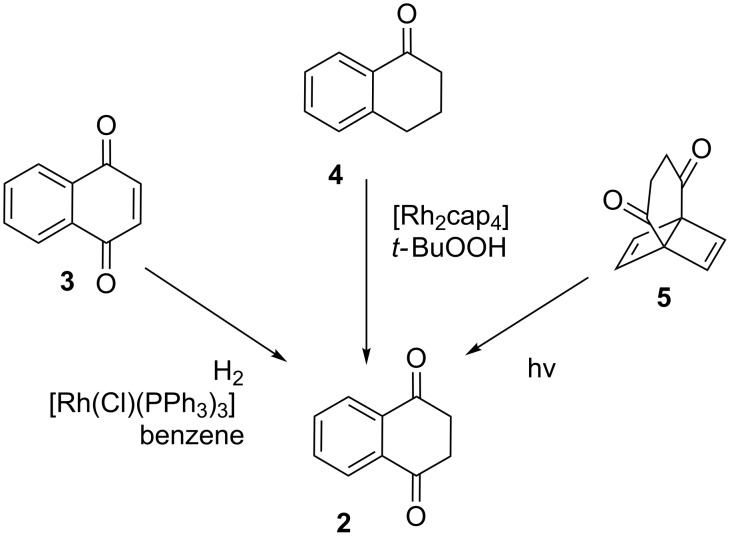
Alternative routes of access to tetralin-1,4-dione.

Tetralin-1,4-dione (**2**) is accessible by tautomerization, reduction, oxidation, and photolytic cycloreversion (Scheme 1 and Scheme 2). Tautomerization takes place upon melting **1** under an inert atmosphere or in a vacuum (>200 °C) [1–3]. The equilibrium mixture at this temperature consists of **1** and **2** in a ratio of 2 : 1 [3]. After cooling to ambient temperature, equilibration ceases and extracts with non-polar solvents are enriched with the more soluble **2**. Tautomerization of **1** was also reported in trifluoroacetic acid, with **2** being the largely dominant species in solution [4].

Tetralin-1,4-dione (**2**) has also been obtained by catalytic hydrogenation of 1,4-naphthoquinone (**3**) using Wilkinson’s catalyst (70% yield) [5], by oxidation of 1-tetralone (**4**) with *t*-BuOOH and a dirhodium caprolactamate catalyst (27% yield at 29% conversion) [6], and by photolysis of the Dewar benzene **5** at low temperature in a solid matrix [7] (Scheme 2).

While dione **2** is readily synthesized, it remains a chemically unexplored curiosity. This simple molecule, and its π-metal complexes, drew our attention and interest for their potential in synthesis. Using the tautomerization of **1** in trifluoroacetic acid to generate **2** [4], we found that upon solvent evaporation the tautomer obtained was dihydroxynaphthalene **1**, rather than diketone **2**. During evaporation, the lower solubility of **1** led to its precipitation and this shifted the equilibrium back. This problem was solved by adding toluene to the mixture before evaporation under vacuum. This, and recrystallization (iPr_2_O) afforded **2** in 72% yield [8]. The straightforward route allowed the synthesis of gram quantities of **2** and the opportunity to study its uncharted chemistry.

This paper details the results of our studies of reductions of the carbonyl functions in diketone **2**.

## Results and Discussion

### Diastereoselective bis-reduction of 2

Reduction of tetralin-1,4-dione (**2**) with a number of reducing agents afforded mixtures of diastereoisomeric *cis*-diol **6** and *trans*-diol **7** in the ratios shown in Table 1. It is important to mention here that these reactions do not occur when tautomer **1** is used.

**Table 1:** Diastereoselective reduction of tetralin-1,4-dione (**2**).

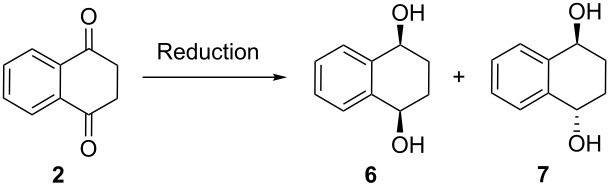			
Entry	Reducing agent^a^	Ratio^b^ **6** : **7**	Yield^c^

1	NaBH_4_	58 : 42	98%
2	LiAlH_4_	32 : 68	94%
3	Red-Al	13 : 87	76%
4	BH_3_·THF	61 : 39	93%
5	L-Selectride	84 : 16	98%

^a^See Supporting Information File 1 for details. ^b^ ^1^H NMR ratios in DMSO-*d*_6_. ^c^Isolated mixture of **6** and **7**.

**Scheme 3:**
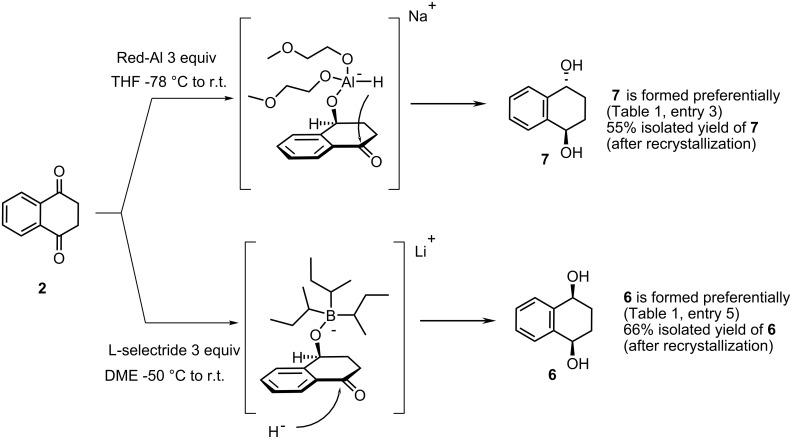
Proposed origin of diastereomeric preference in the reduction of **2**.

The reductions with NaBH_4_ (Table 1, entry 1) and BH_3_·THF (entry 4) gave the diols in high yields but with low diastereoselectivity, slightly favoring the *cis*-diastereoisomer **6**. In contrast, reduction with LiAlH_4_ (entry 2) and, more pronounced, with [Al(H_2_)(OCH_2_CH_2_OMe)_2_][Na] (Red-Al) favored the *trans*-diastereoisomer **7**. Fractional crystallization of the 13 : 87 mixture (entry 3) afforded pure **7** in 55% yield. The reason for the diastereoselectivity in this reaction may have its origin in the delivery of the second hydride from the same aluminium moiety (Scheme 3). Conversely, lithium tri-*sec*-butylborohydride (L-selectride) afforded a product enriched with *cis*-tetralin-1,4-diol (**6**) (entry 5). The high diastereoselectivity presumably is a consequence of the bulky reducing agent. Following the first reduction and formation of the 4-(boranyloxy)-1-tetralone, addition of a second equivalent of L-selectride would be expected to occur from the less hindered face. Hydrolysis then yields preferentially the *cis*-diastereoisomer **6**. The ca. 5 : 1 mixture of **6** and **7** could not be efficiently separated by flash chromatography but recrystallization from iPr_2_O gave *cis*-1,4-dihydroxytetralin (**6**) in 66% yield.

Diols **6** and **7** have been reported previously. They were obtained by treatment of tetralin with NBS to give a 1 : 1 mixture of the corresponding *cis* and *trans*-dibromides, which were converted into diacetates with AgOAc (81% yield). Saponification and fractional recrystallization from MeOH / Et_2_O then afforded pure **6** and **7** though isolated yields were not reported [9]. The *meso*-diol **6** has been used as substrate in enantioselective oxidation [10] and in asymmetric acylation [9,11].

We conclude that while conditions for an efficient highly diastereoselective one-step reduction of both carbonyl functions in **2** have not been realized, enrichment of one or the other diastereoisomer by choice of reducing agent is feasible and acceptable yields of pure diastereoisomers can be obtained.

### Enantioselective bis-reduction of 2

**Scheme 4:**
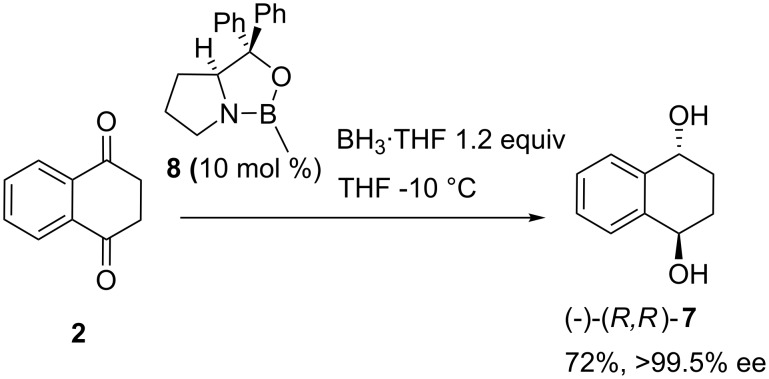
Enantioselective reduction of **2**.

Asymmetric reduction of dione **2** was probed next. This was carried out successfully as shown in Scheme 4 and gave, after two recrystallizations from diisopropylether, (−)-(1*R*,4*R*)-tetralin-1,4-diol (*R*,*R*-**7**) in 72% yield and 99% ee [8]. Only small amounts (ca. 7%) of the *cis* stereoisomer **6** were detected by ^1^H NMR in the crude product. The synthesis of diol **7** in highly enantiomerically enriched form is thus easier than that of the racemate.

The absolute configuration of (−)-(1*R*,4*R*)-**7** agrees with the reliable stereochemical model for the CBS reduction. To our knowledge there is no viable published alternative synthetic access to this *C*_2_ symmetric chiral diol. Compound (−)-(1*R*,4*R*)-**7** was previously obtained by HPLC separation of a 1 : 1 mixture of the *cis*- and *trans*-diols obtained in 55% yield from a four step sequence from (*R*)-1-tetralol [12].

### Mono-reduction of 2

**Scheme 5:**
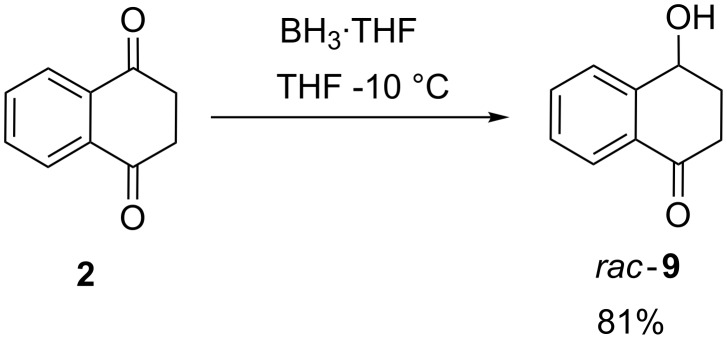
Mono-reduction of **2**.

Mono-reduction was achieved with a reduced amount of borane compared to the reduction detailed above. For the bis reduction, a molar ratio of **2** / BH_3_ of 0.83 was used. Adjusting the ratio to 2.2 (see experimental part) afforded *rac*-**9** in good yield (Scheme 5).

The high yield in mono-reduction is in accord with the expected higher reactivity of the dione **2** compared to the mono-ketone **9**.

### Enantioselective mono-reduction of 2

With an efficient protocol for the synthesis of *rac*-**9** and of *R*,*R*-**7** in hand, research then focused on the more challenging task of enantioselective mono-reduction. First, CBS reduction was performed by slow (1 h) addition of dione **2** to a solution of BH_3_·THF (0.45 equiv) and catalyst **8**. However, background reduction by BH_3_·THF was competitive under these conditions and while (−)-(4*R*)-4-hydroxy-1-tetralone (*R*-**9**) could be isolated in 93% yield, its enantiomeric excess was a modest 53% ee.

**Scheme 6:**
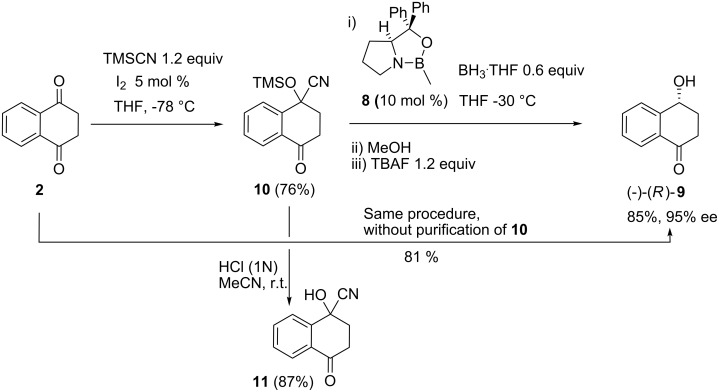
Enantioselective mono-reduction of **2**.

A way to achieve a high ee in mono-reduction was *via* 1-trimethylsiloxy-4-oxotetralin-1-carbonitrile (**10**) as protected equivalent of dione **2**. Slow addition over 2 h of a THF solution of ketone **10** to a solution of BH_3_·THF (0.6 equiv) and catalyst **8** in THF at −30 °C gave, after MeOH quenching and TBAF deprotection, (−)-**9** in 85% yield and 95% ee (Scheme 6).

Cyanohydrin silylether **10** partially hydrolyzes on silica and, as it turned out, isolation of this intermediate is not required and this provided a reliable and efficient sequence to highly enantiomerically enriched **9** (Scheme 5). In the course of this optimization, we also isolated cyanohydrin **11**.

We note literature precedent for procedures for the asymmetric synthesis of **9**. The first involves as the key step kinetic resolution by enzymatic hydrolysis of the corresponding acetate with porcine pancreatic lipase giving (−)-**9** in 47% yield and 95% ee [13]. A second approach uses a Pd-catalyzed asymmetric oxidation of *meso*-tetralin-1,4-diol (**6**) with (−)-sparteine (20 mol %) to give (+)-**9** in 72% yield and 95% ee [10].

We note that chiral 1,4-disubstituted tetralins are of interest in medicinal chemistry. An example is the commercial antidepressant drug sertraline (Zoloft ®) [14–16]. A number of natural products such as preussomerin A [17], catalponol [18], junglanoside A [19], and isoshinanolone [20] contain the 4-hydroxy-1-tetralone unit. 4-Hydroxy-1-tetralone (**9**) itself is a naturally occurring compound isolated from *Ampelocera edentula* with activity against cutaneous leishmaniasis [21]. The straightforward access to highly enantiomerically enriched **9** reported here will be useful.

## Conclusion

Diastereoselective and enantioselective reductions of the unexplored tetralin-1,4-dione provides a very convenient entry into a number of synthetically highly attractive 1,4-tetralindiols and 4-hydroxy-1-tetralone.

## Supporting Information

File 1 Experimental procedures, full spectroscopic and analytical data of compounds **2**, **6**, **7**, **9**–**11**.
