# Supplementary material for: Diastereoselective and enantioselective reduction of tetralin-1,4-dione
Source: Beilstein J Org Chem. 2008 Oct 22;4:37. doi: 10.3762/bjoc.4.37 (PMC2587944; doi:10.3762/bjoc.4.37)
Supplement: File 1 — Experimental procedures, full spectroscopic and analytical data of compounds 2, 6, 7, 9–11. [file Beilstein_J_Org_Chem-04-37-s001.doc]

# Supporting Information

Diastereoselective and enantioselective reduction of tetralin-1,4-dione

E. Peter Kündig* and Alvaro Enriquez-Garcia, Department of Organic Chemistry, University of Geneva, 1211 Geneva 4, Switzerland

[peter.kundig@unige.ch](mailto:peter.kundig@unige.ch)
* Corresponding author

**Experimental procedures, full spectroscopic and analytical data of compounds 2, 6, 7, 9–11.**

**General.** All reactions, except the NaBH_4_ reduction, were carried out under a positive pressure of nitrogen in dry degassed solvents. Glassware was oven dried, and further dried by placing under vacuum and heating with a heat gun for ca. 5 min as necessary. Solvents (THF, diethyl ether, *n*-hexane, toluene and dichloromethane) were purified by using a Solvtek® system. Other solvents were purified by distillation over Na/benzophenone. Commercially available chemicals were used as supplied unless stated otherwise. Flash column chromatography was carried out using silica gel (60 L, 32–63 mesh, Brunschwig SA, Basel). Thin-layer chromatography was performed on pre-coated aluminum plates (Merck silica 60F254), and visualized using UV light, aqueous KMnO_4_ or ceric ammonium molybdate acidic solution. ^1^H, ^31^P and ^13^C NMR spectra were recorded on Bruker AMX 300, 400 and 500 spectrometers. Chemical shifts are quoted relative to tetramethylsilane and referenced to residual solvent peaks as appropriate. Infrared spectra were recorded on a Perkin–Elmer Spectrum One spectrophotometer as neat liquids using a Golden Gate accessory. Optical rotations were measured using a Perkin–Elmer 241 Polarimeter with a Na lamp (589 nm, continuous). LR-MS were acquired using a Varian CH4 or SM1 spectrometer with the ionizing voltage at 70 eV while HR-MS were measured in + TOF mode in the ESI-MS mode using an Applied Biosystems/Scix (Q-STA) spectrometer. HPLC analyses were recorded on a Agilent HP 1100 Series instrument. Microanalysis was carried out by H. Eder, School of Pharmacy, University of Geneva.

**2,3-dihydro-1,4-naphthoquinone (2).** Under N_2_, a 2L round bottom flask was charged with 1,4-dihydroxynaphthalene (**1**) (10 g, 62.4 mmol) [1] and CF_3_COOH (200 mL). The initial suspension changed to a clear solution upon stirring at r.t. for 30 min. Toluene was added (800 mL) and the solvent removed under low pressure at 35 °C. The solid was recrystallized from hot iPr_2_O (400 mL) to afford **2** (7.38 g, 74%) as a white solid. Spectral data agreed with literature values [2-5].

MW = 160.17 g / mol; *R*_F_ = 0.39 (Et_2_O / pentane, 1 : 6); mp = 100–101 °C (iPr_2_O); IR (neat, cm^-1^): 3352, 2971, 2910, 1683, 1585, 1425, 1282, 1200; ^1^H NMR (300 MHz, CDCl_3_): δ 8.05–8.01 (m, 2H), 7.76–7.71 (m, 2H), 3.08 (s, 4H); ^13^C NMR (75 MHz, CDCl_3_): δ 196.2, 135.5, 134.5, 126.9, 37.7; MS *m/z* (EI): 160 (90 [M]^++^), 132 (12), 104 (100), 76 (52), 50 (28); HRMS (ESI) calcd. for C_10_H_9_O_2_ [M+H]^+^: 161.0597 found 161.0603; El. Anal.: calcd. for C_10_H_8_O_2_: 74.99% C, 5.03% H; found 74.78% C, 5.05% H.

**(1*R*,4*S*)-1,2,3,4-tetrahydronaphthalene-1,4-diol (6)** A solution of L-Selectride (1 M in THF, 4.7 mL, 4.68 mmol) was added dropwise under N_2_ to a stirred, cold (−50 °C) solution of tetralin-1,4-dione (**2**) (250 mg, 1.56 mmol) in DME. After 3 h, the reaction mixture was warmed to r.t. and stirred for 1.5 h (TLC control) and then quenched with 10 mL of a 3 N solution of NaOH and 3.9 mL of 30% aq H_2_O_2_. After 30 min stirring, the reaction mixture was extracted with AcOEt (4 x). The combined organic layers were dried over MgSO_4_ and volatiles were removed under reduced pressure. The crude product was taken up in AcOEt / pentane (1 : 1) and filtered through a plug of silica to give, after solvent removal, 248 mg (98%) of a product shown by ^1^H NMR to consist of a 84 : 16 mixture of **6** and **7**. Three recrystallizations from hot iPr_2_O yielded *cis*‑1,4‑tetralindiol (**6**) as white crystals, 166 mg, 66% yield. Spectral data agreed with literature values [6,7].

MW = 164.20 g / mol; *R*_F_ = 0.29 (ether); mp = 137–138 °C (iPr_2_O); IR (neat, cm^–1^): 3257, 2949, 2920, 1486, 1356, 1281, 1196, 1090, 1038; ^1^H NMR (400 MHz, CDCl_3_): δ 7.50–7.45 (m, 2H), 7.34–7.30 (m, 2H), 4.76–4.72 (m, 2H), 2.09–1.98 (m, 4H), 1.79 (broad s, 2H); ^13^C NMR (100 MHz, CDCl_3_): δ 138.9, 128.3, 128.0, 68.3, 28.5; MS *m/z* (EI): 164 (18, [M]^++^), 146 (95, [M–H_2_O]^+^), 131 (30), 120 (100), 105 (50), 77 (48); HRMS (ESI) calcd. for C_10_H_12_O_2_Na [M+Na]^+^: 187.0729, found 187.0734. El. Anal.: calcd. for C_10_H_12_O_2_: 73.15% C, 7.37% H; found 72.96% C, 7.41% H.

**trans-tetrahydronaphthalene-1,4-diol (*rac*-7).** A solution of [Al(H_2_)(OCH_2_CH_2_OMe)_2_][Na] (Red‑Al) (3.5 M in toluene, 1.33 mL, 4.68 mmol) was added dropwise under N_2_ to a −78 °C solution of tetralin-1,4-dione (**2**) (250 mg, 1.56 mmol) in THF (31 mL). After stirring for 2 h, the reaction mixture was warmed to r.t., quenched with saturated aqueous KNaC_4_H_4_O_6_ (Rochelle salt) (30 mL) and stirred for 1 h. The mixture was then extracted with ether (3x), the combined organic phases were dried over MgSO_4_ and volatiles were removed under reduced pressure. The crude product was taken up in AcOEt / pentane (1 : 1) and filtered through a plug of silica to give, after solvent removal, 192 mg (76%) of a product shown by ^1^H NMR to consist of a mixture of **6** and **7** in a ratio of 13 : 87. Recrystallization from hot CHCl_3_ (12 mL) yielded (±)-**7** as a light brown solid. Spectral data agreed with literature values [5-7].

**Reduction of 2 with NaBH_4_:** A Schlenk tube was charged with tetralin-1,4-dione (**2**) (250 mg, 1.56 mmol) and MeOH (39 mL) and cooled to 0 °C. Then, NaBH_4_ (129 mg, 3.43 mmol) was added in small portions. After 30 min of stirring, the reaction was quenched with 10 mL of H_2_O and 10 mL saturated aqueous NaCl. The mixture was extracted with Et_2_O (3 x) and the combined organic phases were dried with MgSO_4_ and volatiles were removed under reduced pressure. The crude product was taken up in AcOEt / pentane (1 : 1) and filtered through a plug of silica to give, after solvent removal, 249 mg (98%) of a product shown by ^1^H NMR to consist of a 58 : 42 mixture of **6** and **7**.

**Reduction with of 2 with LiAlH_4_:** A Schlenk tube was charged with tetralin-1,4-dione (**2**) (250 mg, 1.56 mmol) and THF (15 mL). Then LiAlH_4_ (71 mg, 1.87 mmol) was added in small portions to the stirred cold (−78 °C) solution. After 3 h the reaction was quenched by dropwise addition of AcOEt (10 mL) and warmed up to r.t. A saturated aqueous solution of KNaC_4_H_4_O_6_ (Rochelle salt) (30 mL) was added next. After 1 h the reaction mixture was extracted with Et_2_O (3 x). The combined organic phases were dried over MgSO_4_ and volatiles were removed under reduced pressure. The crude product was taken up in AcOEt / pentane (1 : 1) and filtered through a plug of silica to give, after solvent removal, 238 mg (94%) of a product shown by ^1^H NMR to consist of a 32 : 68 mixture of **6** and **7**.

**Reduction of 2 with borane:** A Schlenk tube was charged with tetralin-1,4-dione (**2**) (250 mg, 1.56 mmol) and THF 22 mL. The mixture was cooled to −10 °C and a solution of BH_3_·THF complex (1 M in THF, 1.87 mL, 1.87 mmol) was added dropwise. After 1.5 h stirring, quenching with 10 mL of MeOH and evaporation of the solvent under reduced pressure afforded a product that was taken up in AcOEt / pentane (1 : 1) and filtered through a plug of silica. Evaporation to dryness gave 235 mg (93%) of a product shown by ^1^H NMR to consist of a mixture of **6** and **7** in a 61 : 39 ratio.

***rac-*4-hydroxy-3,4-dihydronaphthalen-1(2*H*)-one (*rac*-9)*.*** Under N_2_, a solution of BH_3_·THF (1 M in THF, 5.61 mL, 5.61 mmol) was added dropwise to a stirred, cooled (−10 °C) solution of tetralin-1,4-dione (**2**) (2.00 g, 12.5 mmol) in dry THF (120 mL). The clear solution was stirred for 90 min, quenched with MeOH (30 mL) and warmed to r.t. Volatiles were removed under reduced pressure and the residue was purified by flash chromatography (Et_2_O / pentane 1 : 2) to give (±)‑4‑hydroxy-1-tetralone (**9**) as a light-brown oil, 1.65 g, 81% yield. Spectral data agreed with literature values [9,10].

MW = 162.20 g / mol; *R*_F_ = 0.29 (AcOEt / pentane, 1 : 2); IR (neat, cm^-1^): 3404, 2951, 1682, 1601, 1329, 1286; ^1^H NMR (300 MHz, CDCl_3_): δ 7.95 (d, *J* 7.8, 1H), 7.60–7.50 (m, 2H), 7.30–7.45 (m, 1H), 4.98–4.90 (m, 1H), 2.96–2.76 (m, 1H), 2.61(broad singlet, OH, 1H), 2.59–2.49 (m, 1H), 2.42–2.31 (m, 1H), 2.19–2.08 (m, 1H); ^13^C NMR (75 MHz, CDCl_3_): δ 198.1, 145.7, 134.3, 131.2, 128.4, 127.3, 127.2, 67.9, 35.3, 32.2; MS *m/z* (EI): 162 (24 (M+)^+^), 147 (15), 134 (85), 120 (30), 105 (100), 91 (10), 77 (40); HRMS (ESI) calcd. for C_10_H_11_O_2_ [M+H]^+^: 163.0753, found 163.0755. HPLC: Chiralcel OJ, F = 1 mL / min, Hexane / iPrOH 99 : 1, gradient to 90 / 10 over 60 min, T_R_ = 39.5 min [(4*R*)-**9**] and 41.6 min [(4*S*)‑**9**], λ = 254 nm.

**(−)-(*R*)-4-hydroxy-3,4-dihydronaphthalen-1(2*H*)-one ((−)-9).** A solution of ketone (**10**) (200 mg, 0.77 mmol) in dry THF (6 mL) was added under N_2_ with a syringe pump over 1 h to a stirred, cooled (−30 °C) solution of BH_3_·THF (1 M in THF, 462 µL, 0.46 mmol) and (*S*)-3,3-diphenyl-1-methyltetrahydro-3*H*-pyrrolo[1,2-c][1,3,2]oxazaborole (**8**) (21 mg, 0.077 mmol) in dry THF (4.6 mL). After 30 min, the reaction mixture was quenched with MeOH (4 mL) and warmed to r.t. Then, a solution of TBAF (1 M in THF, 925 µL, 0.93 mmol) was added and the mixture stirred for further 30 min. The mixture was washed with sat. aq NaHCO_3_ (3 x) and extracted with Et_2_O (3 x). The combined organic phases were dried over MgSO_4_ and evaporated under reduced pressure. Flash chromatography (AcOEt / pentane 1 : 2) afforded (−)-(*R*)-**9** as pale brown oil, 106 mg, 85% yield, 95.5% ee, [α]_D_^20^**:** −39 (*c =* 0.70, CH_2_Cl_2_). Spectroscopic data see *rac-***9**.

**4-oxo-1-(trimethylsilyloxy)-1,2,3,4-tetrahydronaphthalene-1-carbonitrile (10)**

Under N_2_, a Schlenk tube was charged with tetralin-1,4-dione (**2**) (500 mg, 3.12 mmol), I_2_ (40 mg, 0.16 mmol) and 31 mL of dry CH_2_Cl_2_. The resulting pink solution was cooled to −78 °C and TMSCN (499 µL, 3.74 mmol) was added gently. Stirring was continued for 3 h at this temperature. The reaction was then quenched with aq Na_2_S_2_O_3_ (1.5 N) and shaken in an extraction funnel until decoloration occurred. The aqueous phase was extracted with CH_2_Cl_2_ (3 x). The combined organic phases were dried over Na_2_SO_4_ and the solvent was removed at reduced pressure. Flash chromatography (AcOEt / pentane 1 : 19) of the residue on silica gel gave **10** as a colorless pale green oil, 618 mg, 76% yield.

MW = 259.38 g / mol; *R*_F_ = 0.60 (Et_2_O / pentane, 1 : 2); IR (neat, cm^-1^): 2960, 1691, 1598, 1454, 1412, 1346, 1254, 1105, 1077; ^1^H NMR (300 MHz, CDCl_3_): δ 8.07 (dd, *J* 7.7 and 1.5, 1H), 7.89 (dd, *J* 7.8 and 1.5, 1H), 7.68 (td, *J* 7.7 and 1.5, 1H), 7.54 (td, *J* 7.7 and 1.5, 1H), 2.97 (ddd, *J* 18.0, 8.0 and 5.6, 1H), 2.85 (ddd, *J* 18.0, 8.0 and 5.6, 1H), 2.63–2.61 (m, 2H), 0.2 (s, 9H); ^13^C NMR (75 MHz, CDCl_3_): δ 195.2, 141.2, 134.7, 130.8, 130.5, 128.1, 127.1, 120.4, 69.4, 37.2, 33.8, 1.4; MS *m/z* (EI): 259 (9 [M]^++^), 244 (98), 231 (37), 226 (38), 217(61), 189(58), 75 (100); HRMS (EI) calcd. for C_14_H_17_O_2_NSi [M]^+^: 259.1028 found 259.1024.

**1-hydroxy-4-oxo-1,2,3,4-tetrahydronaphthalene-1-carbonitrile (11)**

A round bottom flask was charged with trimethylsilylcyanohydrin **10** (450 mg, 1.73 mmol), 1.7 mL of MeCN and 1.7 mL of 1 N HCl. This mixture was stirred for 1 h at r.t. and then extracted with AcOEt (3 x). The combined organic layers were dried with MgSO_4_ and evaporated under reduced pressure. Recrystallization from iPr_2_O / pentane, gave **11** as white crystals, 269 mg, 87%.

MW = 187.20 g / mol; mp = 115–117 °C (iPr_2_O / pentane); IR (neat, cm^-1^): 3384, 1682, 1598, 1455, 1411, 1345, 1288, 1195; ^1^H NMR (300 MHz, CDCl_3_): δ 8.07 (d, *J* 7.5, 1H), 7.85 (d, *J* 7.5, 1H), 7.72 (td, *J* 7.2 and 1.5, 1H), 7.58 (td, *J* 7.5 and 1.3, 1H), 3.36 (broad s, 1H), 3.31–2.97 (m, 1H), 2.89–2.76 (m, 1H), 2.73–2.61 (m, 2H); ^13^C NMR (75 MHz, CDCl_3_): δ 195.1, 139.9, 135.1, 130.9, 130.7, 128.2, 126.8, 120.3, 68.2, 35.7, 33.6; MS *m/z* (EI): 187 (100 [M]^++^), 160 (63), 132 (12), 104 (100), 76 (51), 50 (32); HRMS (ESI) calcd. for C_12_H_11_O_2_Na [M+Na]^+^: 210.0656, found 210.0652. El. Anal.: calcd. for C_11_H_9_O_2_N: 70.58% C, 4.85% H, 7.48% N; found 70.58% C, 4.94% H, 7.35% N.

**References**

1. Russig, F. *J. Prakt. Chem.* **1900,** *62,* 30–60. doi:[10.1002/prac.19000620102](http://dx.doi.org/10.1002/prac.19000620102)
2. Birch, A. J.; Walker, K. A. M. *Tetrahedron Lett.* **1967,** 3457–3458. doi:[10.1016/S0040-4039(01)89820-8](http://dx.doi.org/10.1016/S0040-4039(01)89820-8)
3. Pearson, M. S.; Jensky, B. J.; Greer, F. X.; . Hagstrom, J. P.; Wells, N. M. *J. Org. Chem.* **1978,** *43,* 4617–4622. doi:[10.1021/jo00418a017](http://dx.doi.org/10.1021/jo00418a017)
4. Catino, A. J.; Nichols, J. M.; Choi, H.; Gottipamula, S.; Doyle, M. P. *Org. Lett.* **2005,** *7,* 5167–5170. doi:[10.1021/ol0520020](http://dx.doi.org/10.1021/ol0520020)
5. Kündig, E. P.; García, A. E.; Lomberget, T.; Bernardinelli, G. *Angew. Chem., Int. Ed.* **2006,** *45,* 98–101. doi:[10.1002/anie.200502588](http://dx.doi.org/10.1002/anie.200502588)
6. Yamada, S.; Katsumata, H. *J. Org. Chem.* **1999,** *64,* 9365–9373. doi:[10.1021/jo990892p](http://dx.doi.org/10.1021/jo990892p)
7. Boyd, D. R.; Sharma, N. D.; Kerley, N. A.; McConville, G.; Allen, C. C. R.; Blacker, A. J. *ARKIVOC* **2003,** No. vii, 32–48.
8. Joly, S.; Nair, M. S. *Tetrahedron: Asymmetry* **2001,** *12,* 2283–2287. doi:[10.1016/S0957-4166(01)00404-9](http://dx.doi.org/10.1016/S0957-4166(01)00404-9)
9. Ferreira, E. M.; Stoltz, B. M. *J. Am. Chem. Soc.* **2001,** *123,* 7725–7726. doi:[10.1021/ja015791z](http://dx.doi.org/10.1021/ja015791z)
